# Ileal interposition surgery-induced improvement of hyperglycemia and insulin resistance in Goto-Kakizaki rats by upregulation of TCF7L2 expression

**DOI:** 10.3892/etm.2013.998

**Published:** 2013-03-12

**Authors:** XU SUN, MAOMIN SONG, RIXING BAI, SHI CHENG, YING XING, HUISHENG YUAN, PILIN WANG, LISA ZHOU

**Affiliations:** 1 Department of General Surgery, Beijing Tiantan Hospital, Capital Medical University, Beijing 100050, P.R. China;; 2 Department of Biology and Computer Science, Columbia College, Columbia University in the City of New York, New York, NY 10027, USA

**Keywords:** ileal interposition, type 2 diabetes mellitus, T-cell factor 7-like 2, hyperglycemia

## Abstract

The aim of this study was to investigate the effects of ileal interposition (IT) on glucose and insulin resistance (IR) in type 2 diabetic mellitus (T2DM), and the role of T-cell factor 7-like 2 (TCF7L2), formerly known as TCF4, in the downregulation of hyperglycemia following IT. Goto-Kakizaki (GK) rats subjected to IT surgery (GK-IT group), GK rats subjected to sham surgery (GK-Sham group) and Wistar (WS) rats subjected to sham surgery (WS-Sham group) were investigated in this study. Fasting plasma glucose, body weight, food intake per 1 kg body weight, insulin and a homeostasis model assessment of insulin resistance (HOMA-IR) were measured pre- and post-surgery. The rats were euthanized 28 days post-surgery and the pancreas of each rat was dissected. The expression levels of TCF7L2 mRNA and protein were analyzed by quantitative RT-PCR and western blotting, respectively. Our results revealed that IT improved both fasting plasma glucose levels and IR in GK rats by upregulating the expression of the TCF7L2 protein. IT provides a valuable therapeutic option for patients with T2DM. Upregulation of TCF7L2 protein expression may be a possible mechanism underlying the improvement of T2DM following IT.

## Introduction

Type 2 diabetes mellitus (T2DM) has become a significant worldwide health problem. More than 300 million people have diabetes, representing 6% of the global adult population, with seven million more people developing the disease each year (1). T2DM may lead to premature death in both children and adults, and have devastating complications, including amputations, kidney and heart disease. T2DM results from diverse environmental factors, such as viral infection, obesity, chemical poisoning and genetic variants (2). Even mutations in a single gene, such as calpain-10 (CAPN10), sulfonylurea receptor (ABCC8) or the glucagon receptor (GCGR), may result in this disease. Increased morbidity and mortality in T2DM mainly result from long-term microvascular and macrovascular complications. At present, disease progression of T2DM may be prevented by maintaining a healthy lifestyle and medical management. Moreover, the treatment of T2DM requires a combination of drugs, surgery, and dietary and activity modifications, to improve glycemic control and reduce long-term complications (3). Therefore, an effective treatment for T2DM is urgently required.

Of the current treatments for T2DM, Roux-en-Y gastric bypass (RYGB) surgery is considered to be an effective long-term treatment (4–6). The post-RYGB change in gastrointestinal anatomy is reported to be associated with increased postprandial secretion of incretins, peptides secreted by the gut that may improve glucose tolerance and decrease insulin resistance (IR) (7). However, RYGB surgery involves gastric restriction and bypassing the duodenum. By contrast, ileal interposition (IT) surgery has the advantages of no mechanical restriction of meal size, no loss of absorptive surface and no bypass of the foregut (8). The mechanisms by which IT surgery controls plasma glucose are not clear.

WNT signaling is critical for β-cell proliferation and insulin secretion (9). Transcription factor 7-like 2 (TCF7L2), also known as TCF-4, is a transcription factor that functions as a component of the Wnt signaling pathway. This gene is expressed in several tissues, including the gut and the pancreas, and a variant of the protein is linked to an increased risk of developing T2DM. Shu *et al* reported that the reduced levels of TCF7L2 gene expression in T2DM correlated with the down-regulation of receptors for glucagon-like peptide 1 (GLP-1R) and glucose-dependent insulinotropic polypeptide (GIP-R) expression, and impaired β-cell function (10).

In the current study, we used Goto-Kakizaki (GK) rats, a genetic model of T2DM, to investigate whether IT surgery improves glucose tolerance through upregulating the expression of TCF7L2. Our findings have identified a critical gene mediating the treatment of T2DM following IT surgery and thus provides a theoretical basis for the clinical treatment of T2DM.

## Materials and methods

### Animals

Eight-week-old male GK rats were purchased from Shanghai SLAC Laboratory Animal Co., Ltd. (Shanghai, China). The GK rats were randomly assigned to the GK-IT and GK-Sham groups (n=6 for each). Six age-matched Wistar (WS) rats (Shanghai SLAC Laboratory Animal Co., Ltd.) were allocated to the WS-Sham group. All rats were housed in individual cages for ≥7 days prior to surgery. The rats were kept in a climate-controlled room with 12 h light/dark cycle and received a standard chow diet and water *ad libitum*. All animal experimental procedures were approved by the Capital Medical University Institutional Animal Investigation Committee.

### Surgery and post-surgical care

IT surgery was performed as described by Culnan *et al*(11). In the GK-IT group, rats fasted overnight were anesthetized with an intraperitoneal injection of 300 mg/kg chloral hydrate. The ileum was divided 5 cm from the cecum and again at 15 cm, isolating a 10 cm segment of neurovascularly intact ileum on a mesenteric pedicle. The ileum was then anastomosed with interrupted 5-0 silk. The jejunum was then divided 5 cm from the ligament of Treitz and the ileal segment was anastomosed in an isoperistaltic direction with 5-0 interrupted silk sutures. The abdomen was closed thereafter.

Sham surgeries were performed on the GK-Sham and WS-Sham groups. Sham-surgery animals were anesthetized in the same manner as the IT group. Sham surgeries were performed by making transections in the same locations as in the IT-operated animals, but the bowel segments were reattached by anastomosis to their original position.

All animals were housed individually following the surgery. A liquid diet (10% glucose) was administered for the first 2 days, and the rats were then returned to regular chow.

### Body weight and food intake

The body weight and food consumption of the rats were recorded at 7 days pre-surgery and 7, 14, 21 and 28 days post-surgery. The rate of food intake was calculated using the following equation: Food intake rate = daily food consumption (g)/rat body weight (kg).

### Measurement of fasting plasma glucose

The fasting plasma glucose levels of the rats were measured 7 days prior to surgery and 28 days following surgery using a blood glucose meter after 12 h of fasting.

### ELISA

At day 7 pre-surgery and day 28 post-surgery, 0.5 ml blood was collected from the tail vein at 0 or 30 min following glucose gavage (1 g/kg body weight) after 12 h of fasting. Serum was isolated by centrifugation and stored at −80°C until analysis of the insulin concentrations using the ELISA kit (IBL International GmbH, Hamburg, Germany) according to the manufacturer’s instructions.

### IR analysis

The homeostasis model assessment (HOMA) was used to assess IR from fasting plasma glucose and plasma insulin levels as follows: HOMA-IR = fasting plasma glucose (mmol/l) x fasting insulin (mU/ml)/22.5.

### Tissue collection

All rats were sacrificed 28 days post-surgery. The rats were anesthetized with an intraperitoneal injection of chloral hydrate (300 mg/kg) and pancreatic tissue was collected and stored at −80°C for further analysis.

### RNA extraction and quantitative RT-PCR

Total RNA was isolated from pancreatic tissue with TRIzol (Invitrogen Life Technologies, Grand Island, NY, USA; Cat # 15596026), and the first strand of cDNA was synthesized using M-MLV-RTase (Promega Corporation, Madison, WI, USA) according to the manufacturer’s instructions. The resulting cDNA was used for PCR using the SYBR-Green Master PCR mix (Applied Biosystems, Carlsbad, CA, USA) in triplicate. All quantitations were normalized to the endogenous β-actin control. Primers for qRT-PCR were as follows: TCF7L2, forward: GCCTCTCATCACGTACAGCA and reverse: GGATGGGGGATTTGTCCTAC; β-actin, forward: CACCACCATGTACCCTGGCA and reverse: GCTGTCACCTTCACCGTTCC. PCR and data collection were performed on the TP800 qPCR System (Takara Bio, Inc., Otsu, Japan). The relative quantitation value for the TCF7L2 gene was expressed as 2^−(Ct−Cc)^, where Ct and Cc are the mean threshold cycles of the calibrator and target, respectively, after normalizing to β-actin.

### Western blotting

Total protein was obtained from the pancreatic tissues. Cell lysates (30 *μ*g protein) were resolved on 10% SDS-PAGE and transferred to a polyvinylidene difluoride membrane (Millipore, Bedford, MA, USA). Protein was probed for rabbit anti-TCF7L2 (Abcam, Inc., Cambridge, MA, USA) or rabbit anti-actin (Saier Biotechnology Co., Ltd., Tianjing, China) antibodies, followed by incubation with an HRP-conjugated secondary antibody (Saier Biotechnology Co., Ltd.) and visualized using a Western Lightning^®^ Plus enhanced chemiluminescence substrate (ECL; PerkinElmer Inc., Waltham, MA, USA). The density of the bands was analyzed using LabWorks™ 4.0 (UVP, Upland, CA, USA).

### Statistical analysis

The data were analyzed using SPSS 13.0 software (SPSS, Inc., Chicago, IL, USA) and presented as the means ± SD. Statistically significant differences were determined using one-way analysis of variance (ANOVA). P<0.05 was considered to indicate a statistically significant difference.

## Results

### Effects of IT on rat body weight and food intake

Following surgery, all rats survived the 28-day experiment. No significant differences were observed in body weight and food intake per 1 kg body weight among the three groups pre-surgery. However, two weeks post-surgery, the body weight and food consumption of rats in the GK-IT group were significantly lower than those in the other two groups (P<0.05; Tables I and II).

### Fasting plasma glucose

The fasting plasma glucose levels of the GK-IT and GK-Sham groups were higher than that of the WS-Sham group pre-surgery. The fasting plasma glucose in the GK-IT group after surgery showed a significant reduction when compared with the GK-Sham group (P<0.05), and had no apparent difference compared with the WS-Sham group at 4 weeks post-surgery (Table III).

### Insulin levels

There were no significant differences in fasting plasma insulin levels among all groups pre- and post-surgery. However, 30 min after the intragastric administration of glucose, the insulin levels in the GK-IT and GK-Sham groups were significantly lower than that of the WS-Sham group pre-surgery (Table IV, P<0.05). There was a significant increase in the postgavage insulin levels in the GK-IT group compared with the GK-Sham group after surgery (P<0.05), although the postsurgical insulin level of the GK-IT group remained lower than that of the WS-Sham group (P<0.05). In the GK-IT group, the postgavage insulin levels were significantly higher than the corresponding pre-surgical levels (P<0.05).

### IT improves IR

To investigate whether IT improves IR in GK rats, the HOMA-IR value was examined. As shown in Table V, HOMA-IR values in the GK-IT and GK-Sham groups were significantly higher than those in the WS-Sham group pre-surgery (P<0.05). There was no significant difference in the HOMA-IR values between the GK-IT and WS-Sham groups on day 28 post-surgery, which were both less than that of the GK-Sham group (P<0.05). Post-surgery values of HOMA-IR were higher than pre-surgical values in the GK-Sham group (P<0.05). These results showed that IT improves IR in GK rats.

### IT surgery upregulates the expression of TCF7L2

**Figure 1:**
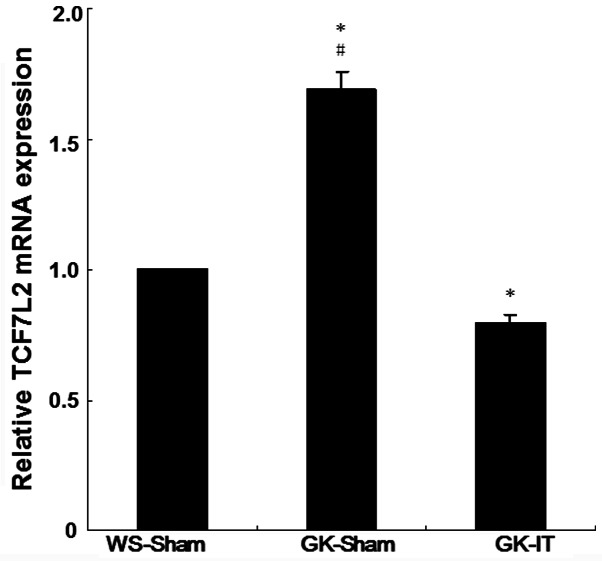
Relative expression levels of TCF7L2 mRNA in the pancreatic tissue among the WS-Sham, GK-Sham and GK-IT groups at 28 days post-surgery. The mRNA levels were analyzed by qRT-PCR and normalized to the expression of actin. Results were presented as the means ± SD of three independent experiments.^*^P<0.05 compared with the WS-Sham group, ^#^P<0.05 compared with the GK-IT group. TCF7L2, T-cell factor 7-like 2, formerly known as TCF4.

To determine the effect of IT on TCF7L2 expression, we first investigated the expression of TCF7L2 mRNA in pancreatic tissue 28 days post-surgery using qRT-PCR. The TCF7L2 mRNA levels in the GK-Sham group were 1.69-fold higher than those of the WS-Sham group, with a statistically significant difference (Fig. 1, P<0.05), suggesting that the expression level of TCF7L2 mRNA was higher in diabetic rats than in normal rats. Following IT surgery, the relative expression levels of TCF7L2 mRNA in GK-IT rats were 20% lower than those of the WS-Sham group, a significant difference (P<0.05). These results indicate that IT surgery decreases the level of TCF7L2 mRNA in the diabetic rats.

**Figure 2:**
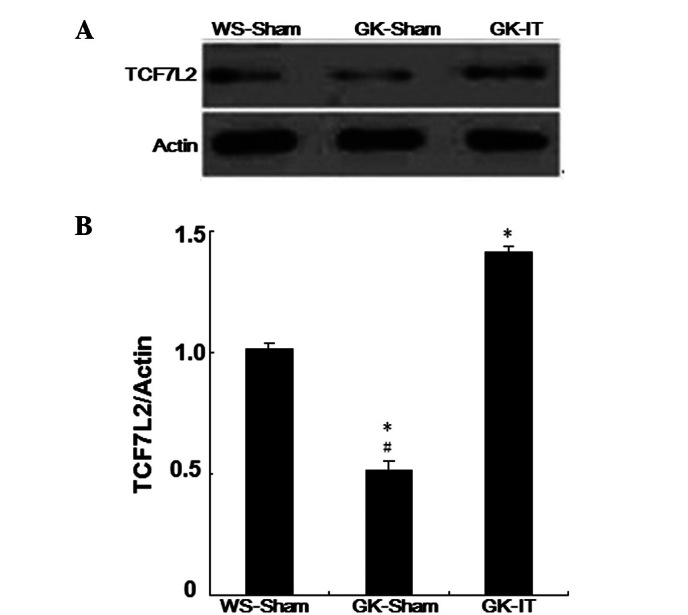
TCF7L2 expression levels in the pancreatic tissue of GK rats. (A) At 28 days after surgery, TCF7L2 expression in rat pancreatic tissues was examined by western blotting. Actin was used as loading control on the same membrane after stripping. (B) Densitometric analysis of results in (A) after normalization to actin. The results presented are the means ± SD of three independent experiments. ^*^P<0.05 compared with the WS-Sham group. ^#^P<0.05 compared with the GK-IT group. TCF7L2, T-cell factor 7-like 2, formerly known as TCF4.

The expression of TCF7L2 protein was analyzed by western immunoblotting. Notably, an increased TCF7L2 protein level was observed in the pancreatic tissues of GK rats following IT surgery (P<0.05 vs. the WS-Sham and. GK-Sham groups; Fig 2). Moreover, the levels of TCF7L2 protein expression in the GK-Sham group were significantly lower than those of the WS-Sham group after surgery (P<0.05; Fig. 2). These findings demonstrate that IT surgery is able to increase the levels of TCF7L2 protein in diabetic rats.

## Discussion

In T2DM, deficiency of insulin production and a decrease in β-cell mass may be attributed to a complex interplay between genetic predisposition and environmental factors. Many studies that have focused on the influence of IT on T2DM rats have discovered that following IT, the rats did not regain weight post-surgery at the same rate as rats that underwent a sham surgical procedure (12–14). Moreover, rats with IT have been able to maintain weight loss and reduce food intake for as long as 6 months after surgery (8). Reduced calorie intake following IT surgery may be one cause of the significantly decreased body weight in the GK-IT group. We discovered that concentrations of fasting plasma glucose and HOMA-IR dropped to normal levels in the GK-IT group following surgery. Although the energy intake and body weight in the GK-IT group post-surgery was increased, the decreased plasma glucose levels did not reverse. The result indicated that energy intake and body weight were not direct reasons for the reduced glucose levels.

In our study, postgavage insulin levels in the GK-IT group increased significantly and there were no differences in fasting plasma insulin levels among the three groups pre-surgery. Previous studies have demonstrated that IT surgery improves islet structure and increases pancreatic insulin content (15,16). The increased insulin levels in the GK group after IT are suggestive of the promotion of β-cell surival and function, since pancreatic β-cells are the only source of insulin production.

The WNT signaling pathway is known to be associated with developmental processes such as embryogenesis, pancreatic islet proliferation and tumorigenesis (17,18). TCF7L2 plays an important role in the downstream signals of the WNT pathway (19). Common genetic variations in the gene that encodes TCF7L2 reveal a strong association of this protein with T2DM (20,21), and a number of studies on T2DM have focused on this transcription factor. It has been shown that, in rodent and human islets, TCF7L2 is essential for normal β-cell survival and secretory functions (22). Shu *et al* reported that silencing the formation of all TCF7L2 isoforms through siRNAs affects the capacity for insulin secretion in rodents (10). *In vitro*, TCF7L2 depletion in islets has been reported to reduce proliferation, induce β-cell apoptosis and reduce glucose-stimulated insulin secretion (22). A recent study has confirmed that the selective deletion of TCF7L2 in the mouse pancreas impairs insulin release and glucose homeostasis (23). In turn, the overexpression of TCF7L2 protects β-cells from apoptosis induced by chronically elevated glucose and cytokines.

In our study, the levels of TCF7L2 mRNA in the GK-IT group were markedly lower than those in the GK-Sham group at 28 days post-surgery, while the protein levels of TCF7L2 were higher. It has been suggested that IT may result in an increase in the expression of TCF7L2 in GK rats. Our results are consistent with those of Shu *et al*(10), and showed that TCF7L2 mRNA and protein levels are reciprocally changed in the islets of rodents with T2DM. The mechanism underlying the difference in regulation of transcription and translation remains unclear. It has been speculated that post-transcriptional regulation of TCF7L2, not the changes in mRNA levels, may be involved in TCF7L2-regulated β-cell function and survival. Therefore, increased TCF7L2 mRNA expression in diabetes may be a consequence of impaired β-cell function owing to a deficiency of TCF7L2 protein. We considered TCF7L2 to be key in the downregulation of plasma glucose after IT.

In conclusion, our study illustrated that IT efficiently down-regulates plasma glucose levels, improves IR, and increases postgavage insulin levels in T2DM. This type of surgery improves TCF7L2 protein expression in pancreatic tissues. Therefore, upregulation of TCF7L2 may be a potential mechanism by which changes are mediated following IT. Further studies are required to detect whether increased TCF7L2 protein levels correlate with upregulation of the GIP receptor and the GLP-1 receptor, which are important for pancreas and β-cell survival, after IT in T2DM.

## Figures and Tables

**Table I:** Rat body weight (g) 1 week prior to and at each of the 4 weeks after surgery in each group.

Time point	GK-Sham	GK-IT	WS-Sham
1 week pre-surgery	242.33±2.73	240.83±3.31	240.67±4.32
1 week post-surgery	233.50±3.08	232.83±3.66	231.50±6.41
2 weeks post-surgery	239.33±2.50	222.50±3.39^a,b^	236.67±5.89
3 weeks post-surgery	247.50±4.97	234.33±3.61^a,b^	249.00±2.10
4 weeks post-surgery	255.33±3.08	239.83±4.99^a,b^	267.67±3.08

a P<0.05 compared with the GK-Sham group,

b P<0.05 compared with the WS-Sham group. Data are expressed as means ± SD.

**Table II:** Food intake rate (g/kg/d) 1 week prior to and at each of the 4 weeks after surgery in each group.

Time point	GK-Sham	GK-IT	WS-Sham
1 week pre-surgery	66.78±1.23	67.02±2.27	67.50±2.34
1 week post-surgery	52.94±2.29	52.30±1.46	54.07±1.15
2 weeks post-surgery	59.61±1.49	40.08±3.53^a,b^	58.78±5.17
3 weeks post-surgery	65.09±2.63	46.08±2.55^a,b^	64.05±3.08
4 weeks post-surgery	73.86±2.51	51.98±2.70^a,b^	77.78±2.64

a P<0.05 compared with the GK-Sham group,

b P<0.05 compared with the WS-Sham group. Data are expressed as means ± SD.

**Table III:** Fasting plasma glucose levels (mg/dl) pre-surgery and 4 weeks post-surgery in each group.

Group	Pre-surgery	4 weeks post-surgery
GK-IT	123.84±7.02^a^	95.40±6.48^b^
GK-Sham	119.34±4.86^a^	130.86±9.54
WS-Sham	92.34±4.32	88.74±6.84^b^

a P<0.05 compared with the WS-Sham group,

b P<0.05 compared with the GK-Sham group. Data are expressed as means ± SD.

**Table IV:** Plasma insulin levels (ng/ml) pregavage and 30 min postgavage in each group.

	Pre-surgery		4 weeks post-surgery	
Group	Pregavage	Postgavage	Pregavage	Postgavage
GK-IT	1.18±0.09	2.84±0.07^a^	1.12±0.07	4.26±0.15^a,b,c^
GK-Sham	1.19±0.08	2.81±0.08^a^	1.19±0.08	2.90±0.12^a^
WS-Sham	1.09±0.03	5.80±0.24	1.14±0.11	5.62±0.28

a P<0.05 compared with the WS-Sham group,

b P<0.05 compared with the GK-Sham group,

c P<0.05 30 min postgavage insulin concentrations post-surgery compared with pre-surgery in the GK-IT group. Data are expressed as means ± SD.

**Table V:** HOMA-IR changes before and after surgery in each group.

Group	Pre-surgery	4 weeks post-surgery
GK-IT	7.61±0.20^a^	5.58±0.12^b,c^
GK-Sham	7.43±0.58^a^	8.09±0.25^c^
WS-Sham	5.28±0.18	5.28±0.18^b^

a P<0.05 compared with the WS-Sham group,

b P<0.05 compared with the GK-Sham group,

c P<0.05 compared with corresponding pre-surgery values. Data are expressed as means ± SD. HOMA-IR, homeostasis model assessment of insulin resistance.
